# Micro-computed tomographic evaluation of the shaping ability of three nickel-titanium rotary systems in the middle mesial canal of mandibular first molars: an ex vivo study based on 3D printed tooth replicas

**DOI:** 10.1186/s12903-024-04024-z

**Published:** 2024-03-02

**Authors:** Qi Zhu, Chao Liu, Bingbing Bai, Fan Pei, Ying Tang, Weijian Song, Xiuchun Chen, Yongchun Gu

**Affiliations:** 1 Department of Dentistry and Central Laboratory, Ninth People’s Hospital of Suzhou, Soochow University, Ludang Road 2666#, Wujiang Dist., Suzhou, 215200 China; 2 The Stomatology Hospital Affiliated of Suzhou Vocational Health College, Renmin Road 829#, Gusu Dist, Suzhou, 215002 China; 3 Department of Pathology, Ninth People’s Hospital of Suzhou, Soochow University, Ludang Road 2666#, Wujiang Dist., Suzhou, 215200 China; 4 Department of Stomatology, Suzhou Municipal Hospital, Daoqian St. 26#, Gusu Dist, Suzhou, 215002 China

**Keywords:** Mandibular first molar, Middle mesial canal, Three-dimensional printing, Root canal instrumentation, Nickel-titanium rotary system

## Abstract

**Background:**

The preparation of the middle mesial (MM) canal of mandibular molars represents a challenge because it is often curved, narrow, and close to the root concave. The purpose of this study was to evaluate the ex vivo shaping ability of 3 nickel-titanium (NiTi) rotary systems in the MM canal using 3D printed resin tooth replicas.

**Methods:**

A permanent mandibular first molar with a MM canal was acquired from a pool of extracted teeth and reproduced by a 3D printer. The resin tooth replicas (*n* = 18) were equally assigned to 3 groups for the evaluation of the shaping abilities of 3 NiTi rotary systems (OneShape [OS], Twisted Files [TF], and ProTaper Gold [PTG]) according to the manufacturer’s recommendations. The tooth replicas were scanned by micro-computed tomography (micro-CT) twice before and after instrumentation of the mesiobuccal (MB), mesiolingual (ML), and MM root canals. After 3D reconstruction, the canal straightening, change of root canal volume and surface area, the mesial and distal canal wall thickness and canal transportation at the levels of 1, 2, and 3 mm below furcation were assessed. One-way variance analysis and Turkey’s post hoc test were used for comparisons of the means among different groups, and paired-*t* test was used to compare the mesial and distal sides of the mesial roots.

**Results:**

As compared with OS and TF, the use of PTG in preparation of MM canals resulted in significantly more straightening of canal curvature (*p* < 0.05), greater post-instrumentation canal volume and surface area, and thinner mesial and distal remaining canal wall thickness at 1, 2 and 3 mm below furcation (all *p* < 0.05). Regarding the root canal transportation in the mesiodistal direction, there was no significant difference among the 3 instruments (all *p* > 0.05) after the preparation of the MB and ML canals. However, in the MM canal, more pronounced transportation was detected in the PTG group at 2 mm below furcation, and in the TF group at 3 mm below furcation as compared with the other 2 systems (both *p* < 0.05).

**Conclusions:**

3D printed tooth replicas have the advantages of consistency and can be an ideal model to evaluate the shaping ability of different instruments in the MM canal. OS and TF files performed similarly and both are appropriate for shaping the MM canal, while PTG may cause excessive and uneven resin removal, especially near the furcation, and may lead to root fragility and procedural errors.

## Background

Thorough cleaning, shaping, and obturation of the entire root canal space are critically important steps for successful endodontic treatment [1]. However, a complex root canal system is often identified in the mesial root of mandibular molars. These anatomic variations could be isthmuses, fins, accessory canals, splitting and/or merging canals at different root levels, which may pose an endodontic challenge [2]. In recent decades, the presence of a middle mesial (MM) canal has constantly been reported and the incidence ranged widely from 1 to 25% in different studies [3–6]. The discrepancy may be due to the different definitions of the MM canals, the diverse methodologies and techniques of detection, and the different ethnic backgrounds of the subjects. The MM canal usually has a small orifice deep within the isthmus or a developmental groove between the orifices of the mesiobuccal (MB) and mesiolingual (ML) canal, and it either joins other canals or shows a separate and distinct canal. Pomeranz et al. [7] classified MM canals into 3 categories: fin, confluent, and independent. In regard to the distance of the apical foreman to the anatomic apex, the MM showed the greatest deviation compared with the MB and ML canals [8]. The variable configurations of MM canals were age-related due to the deposit of secondary dentin [9]. Because the MM canal is located between the MB and ML canals at the sagittal plane of the mesial root, it is prone to be overlooked on conventional intraoral radiographs; while the inability to locate, or improper handling of this additional canal may cause persistent canal infection and treatment failure.

With regard to root canal therapy of mandibular molars, another challenge is associated with the intrinsic canal curvature in the mesial root. The mesial root always curves distally, and several studies demonstrated that the internal root canals were close to the distal surface of the root concavity [10, 11]. Clinicians need to be aware of this danger zone and avoid strip perforation during root canal instrumentation [12]. During curved canal preparation, the traditional stainless-steel files may generate a series of procedural errors, such as ledging, zipping, canal transportation, striping, etc., due to their inherent stiffness and tendency to straighten a curved canal [13]. In recent decades, a series of novel nickel-titanium (NiTi) rotary systems have been developed and widely applied in endodontics. Due to their super-elasticity and increased flexibility, they can significantly improve shaping efficacy and reduce iatrogenic procedural errors.

Twisted Files (TF; SybronEndo, Orange, CA, USA), a multi-file NiTi rotary system developed in 2008, are manufactured with a twisted shape that gives a triangular cross-section. The R-phase heat treatment, twisting of the metal, and special surface conditioning of the instruments can significantly increase their resistance to cyclic fatigue and flexibility, and allow for maintenance of the original canal curvature and minimization of the transportation, even in severely curved canals [14]. ProTaper Gold (PTG; Dentsply Sirona, Ballaigues, Switzerland) has the same geometry as that of ProTaper Universal (PTU), and they are rotary sequential systems with a convex triangular cross-section and a progressive taper. PTGs are manufactured with proprietary gold heat treatment, which can reduce machining process defects and modify the crystalline phase structure. The PTGs demonstrated a 2-stage specific transformation behavior and high austenite finish temperatures, so that they have greater flexibility and resistance to cyclic fatigue [15]. OneShape (OS; MicroMega, Besançon, France) is a single-file system used in continuous rotation, and it show advantages in shortening the instrumentation time and reducing cross-contamination risk [16]. OS consists of a size 25/6% file with a passive tip and a variable cross-section. [16]. Although OS is manufactured with conventional NiTi alloy and is not heat treated, it has been reported that it caused only minimum canal transportation in severely curved canals or S-shaped canals [17, 18].

During preparation of a complex root canal, it is essential for clinicians to understand the features of different NiTi instruments to best meet the anatomic challenges of root canals. It has been previously reported that TF [14], PTG [19] and OS [18] performed well in mesial canals of mandibular molars, and each of them respected the original curvature and was safe to use. However, to the best of our knowledge, no previous studies have evaluated the shaping ability of the above-mentioned 3 NiTi rotary systems in the MM canal.

In recent years, three-dimensional (3D) printing, a novel technology that can quickly and precisely fabricate duplicate physical models, has been applied in endodontics [20, 21]. Based on micro-computed tomography (CT) or cone-beam CT (CBCT) images, the digital tooth models can be constructed three-dimensionally and saved in standard tessellation language (STL) format. The STL files can be input into a 3D printer to duplicate replicas of teeth with high resolution. Because 3D printed tooth models are easy to obtain and similar in anatomy, more and more scholars recently utilized 3D printed teeth for the study of root canal preparation [22–24]. The purpose of this study was to evaluate the ex vivo shaping ability of 3 different NiTi rotary systems in the MM canal based on 3D printed resin tooth replicas and micro-CT analysis.

## Materials and methods

### Collection of sample teeth

Ethnic approval of the use of extracted human teeth was obtained from the Committee in Ethics and the institutional review board (Issuing Number: KY2022-089-01) of the Ninth People’s Hospital of Suzhou. All subjects were native Chinese, and the teeth were extracted because of periodontal disease, nonrestorable caries, trauma, or prosthodontic reasons. The exclusion criteria were as follows: (a) teeth with open root apexes, (b) teeth display visible apical resorption, (c) teeth have been endodontically treated previously. A total of 45 permanent mandibular first molars were included in the current study.

### Micro-CT scanning of extracted teeth

Each tooth was scanned along the tooth axis using micro-CT scanning (SkyScan1174; Bruker-microCT, Kontich, Belgium) at a 17-µm voxel size, 800 mA, rotation step of 0.7˚, 50 kVp and 1- mm thick aluminum filter, 1 frame averaging and arch rotation of 180˚. The micro-CT data sets were then transferred to the Mimics 21.0 (Materialise, Leuven, Belgium) software to perform 3D reconstruction of the teeth and root canal systems.

The root canal configurations in the mesial root were examined and only one case of MM canal was representative and selected for 3D printing. The MM canal left the pulp chamber with its own orifice, and jointed to the ML canal about 1 mm short of the apex.

### 3D printing of the mandibular first molar with an MM canal

In Mimics software, the tooth crown was virtually removed at 2 mm above the cemento-enamel junction (CEJ) to entirely expose the pulp floor and root canal orifices. Then the digital tooth model was saved in standard tessellation language (stl) format and exported to a Saturn 2 3D printer (ELEGOO, China) at a resolution of XY: 28.5 μm and Z: 20 μm performed by MOLEGRID™ Ultradetail photopolymer resin printing material (KEXCELLED, China).

### Root canal instrumentation

A total of 18 tooth replicas were used and randomly assigned to 3 experimental groups (*n* = 6 per group). The MB, ML, and MM canals were prepared according to the manufacturer’s recommendations for each NiTi rotary system. The root canals were negotiated with size #10 and #15 K-file (Dentsply Maillefer, USA) until the tip was visible at the apical foramen. The working length (WL) was determined as 1 mm short of the foramen. After the WL determination, a single operator (the first author), who had been specially trained with all 3 NiTi systems, performed the instrumentation in all groups. The NiTi files were driven by the electric motor (X-smart Plus, Dentsply Maillefer, USA) in a continuous clockwise rotation, and each NiTi file was used in only 1 canal. The debris on the files was cleaned with wet gauze, and the canal was irrigated with 2 mL of distilled water each time.

- Group OS: OS files (25/6%) were used at the WL with a rotational speed of 400 rpm, and the torque was adjusted to 4 Ncm.
- Group TF: Canals were instrumented at a speed of 350 rpm and 2.5 Ncm torque in crown-down fashion. The 25/8% was used to prepare the coronal third, and 25/6% was inserted and used until 2 mm short of the working length (WL). Then 25/4% and 30/4% were used sequentially to WL.
- Group PTG: PTG instruments were used in a crown-down fashion. SX, S1, and S2 files are for the preparation of the coronal and middle thirds, and F1, F2, and F3 are for the preparation of the apical third. The SX was used for cervical preparation. Then, the S1 (17/2%), S2 (20/4%), F1 (20/7%), F2 (25/8%), and F3 (30/9%) were used sequentially to WL at 300 rpm and 2 Ncm torque.

**Fig. 1:**
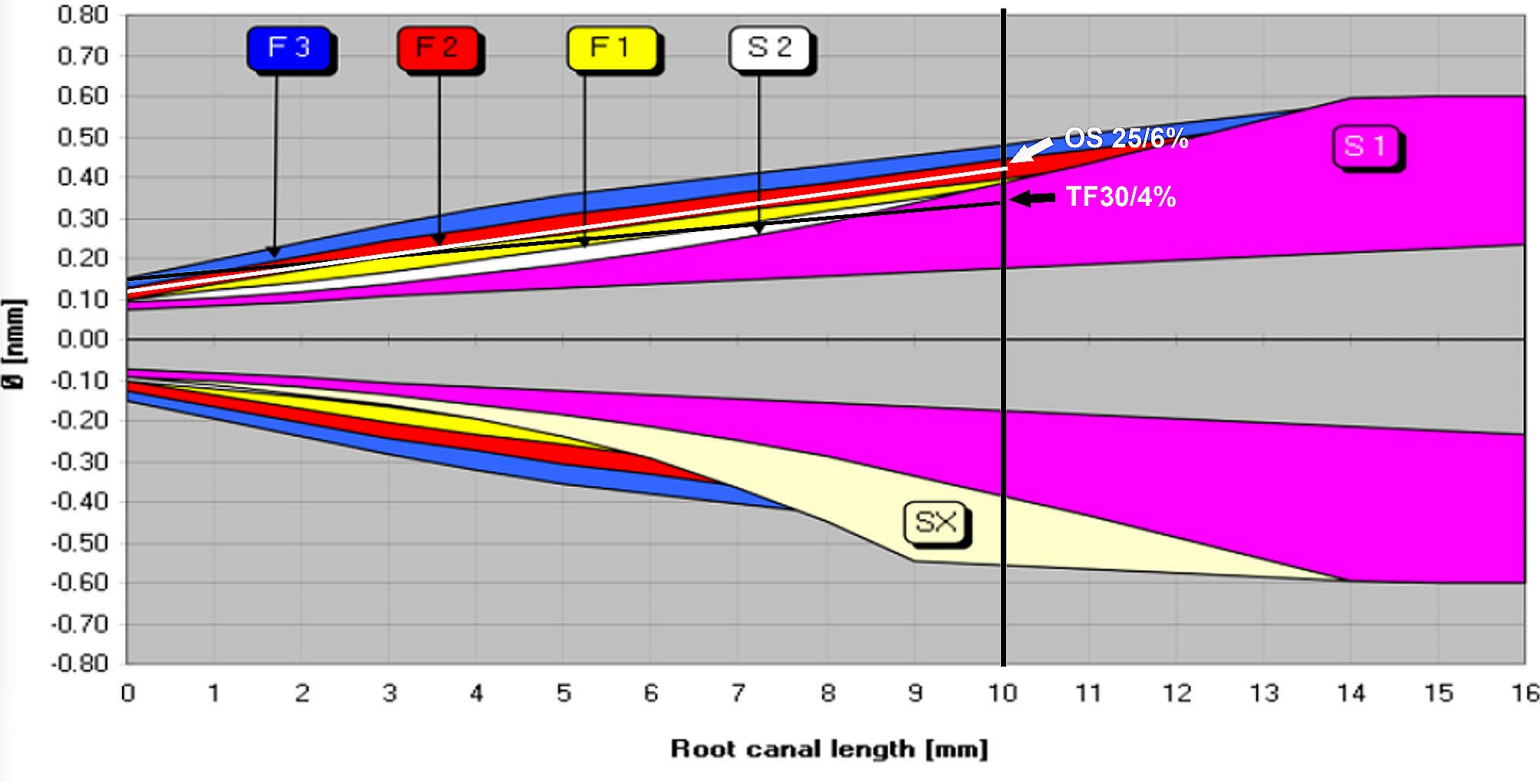
Diagram of the progressive taper design (ProTaper Gold system) and constant taper design (Twisted File and OneShape system) of 3 NiTi instruments. The white arrow is OS 25/6% (D_0_ = 0.25 mm, D_10_ = 0.85 mm) and the black arrow is TF 30/4% (D_0_ = 0.30 mm, D_10_ = 0.70 mm); the 2 lines intersect at the level (D _2.5_ = 0.40 mm) according to calculation

The diagram of the progressive taper design (PTG) and constant taper design (TF and OS) of the 3 NiTi instruments is shown in Fig. 1. The use of NiTi instruments did not cause strip perforation or instrument separation in any groups.

### Micro-CT imaging and analysis of tooth replicas

Each tooth replica was scanned twice using micro-CT before and after root canal preparation following the same scanning and reconstruction protocols. No instrument separation or strip perforation occurred during the instrumentation process.

**Fig. 2:**
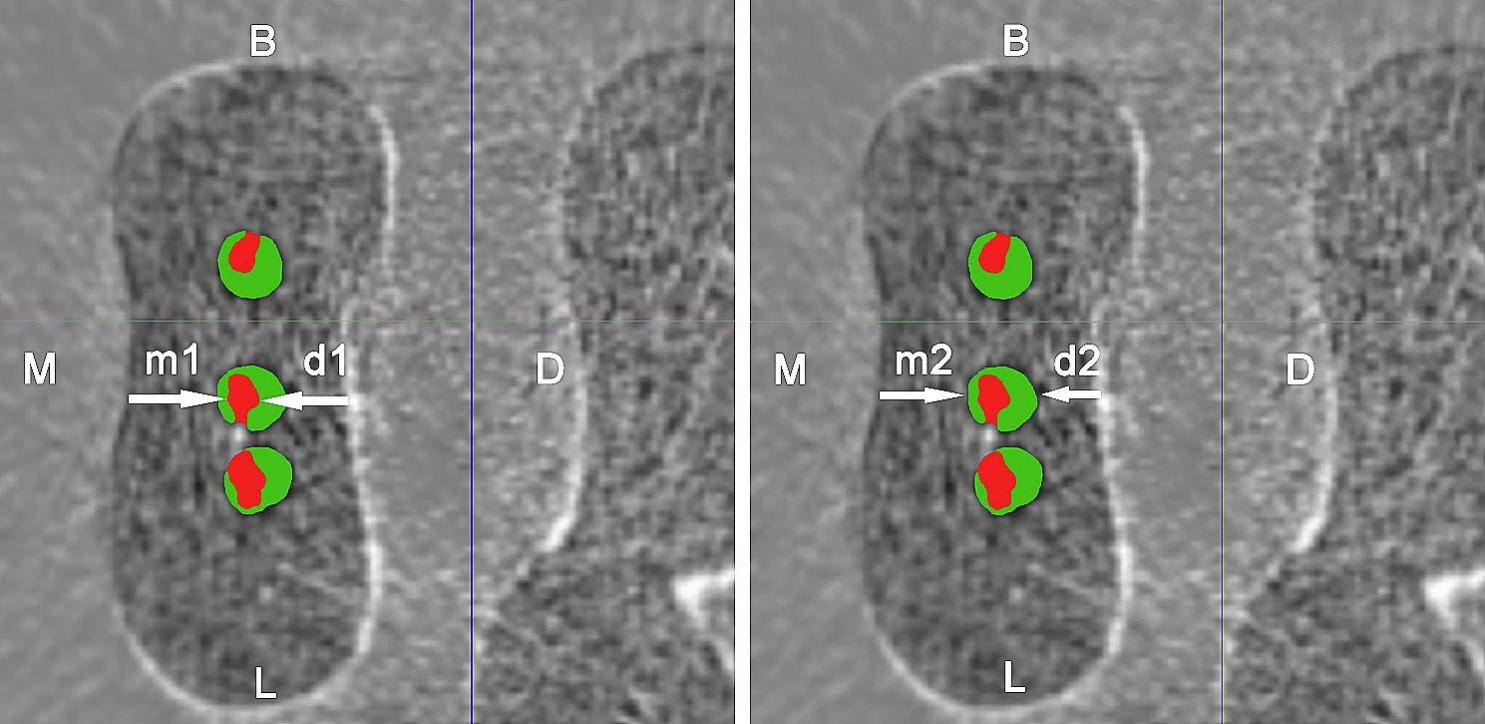
Measurement of the mesial and distal canal wall thickness before and after instrumentation in the axial micro-CT images (1 mm below furcation). Instrumentation with OneShape caused distal transportation of the MM canal. m1 and d1 are mesial and distal canal wall thickness pre-instrumentation, respectively; m2 and d2 are mesial and distal canal wall thickness post‑instrumentation, respectively (red: preoperative canal shape, green: postoperative canal shape)

**Fig. 3:**
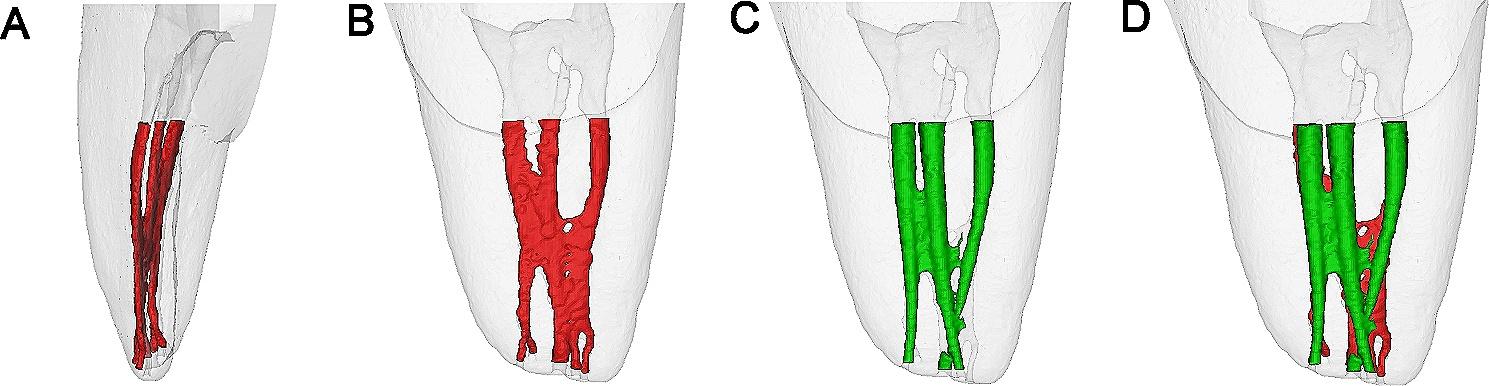
Representative micro-CT images of a 3D printed mandibular first molar tooth replica with an MM canal (3D reconstruction and superimposition of the root canal system in the mesial root before and after instrumentation). **A** The mesial root curved distally (before instrumentation, a clinical view); **B** the root canal configuration in the mesial root before instrumentation (a proximal view); **C** the root canal configuration in the mesial root after instrumentation with OneShape (a proximal view); **D** superimposition of the root canal configurations in the mesial root pre-(red) and post-instrumentation (green)

Pre- and post-operative images were superimposed using the 3D registration function of the software DataViewer v.1.5.1 (Bruker-microCT, Kontich, Belgium) (Fig. 2), and Mimics software was used to calculate the quantitative morphologic parameters and construct visual 3D models of the mesial root and root canal system (Fig. 3). The geometric parameters evaluated were as follows:

#### Root canal length

Along the MM canal from the orifice to the apical foreman (AF), 10–20 center points of root canal cross-sections were defined and linked to create a spline curve. The length of the spline could be obtained directly via the software and taken as the whole canal length. The canal length from the furcation level to the AF was also obtained.

Root canal straightening: In the plane of canal curvature (mostly in the coronial plane), the angles of the root canal curvature pre- and post-instrumentation were measured according to Schneider’s method [25]. The decrease in the degrees of canal curvature after instrumentation was calculated to quantify the canal straightening.

#### The width of the MM canal at the WL

The long and short diameters of the MM canal were measured at the cross-section 1 mm short of the apical foramen.

#### Root canal volume and canal surface

The volume of interest for each specimen was determined from the furcation level to the anatomical apex of the mesial roots. Root canal volume and surface area of MB, ML and MM canals pre- and post-instrumentation were obtained. The percentage of volume/surface area increase (% ∆) was obtained through a formula that included data before (B) and after (A) canal preparation:

$$\it \it \it \normalsize \tt \it \it \% \,\Delta \, = \,\left( {\left[ {{\rm{A}} - {\rm{B}}} \right]\,/\,B} \right)\, \times \,100$$

Canal wall thickness and canal transportation in the mesiodistal direction: Measurements of minimum canal wall thickness pre- and post-instrumentation for MB, ML and MM canals were performed as reported by Lim et al. [12] Canal transportation at 3 cross-section levels corresponding to 1, 2, and 3 mm below furcation for each NiTi system was calculated by the following formula: Canal transportation = (d1-d2) – (m1-m2), where d1 and d2 were the pre- and post-instrumentation minimum distal canal wall thickness, respectively, and m1 and m2 were the pre- and post-instrumentation minimum mesial canal wall thickness, respectively (Fig. 2).

### Statistical analysis

One-way analysis of variance (ANOVA) and Turkey post hoc test were used for multiple comparisons among groups. Paired *t*-test was used to compare the mean canal wall thickness between the distal and mesial sides. The significance level was set at 5%.

## Results

### The homogeneity of tooth replicas before instrumentation

The mean length of the MM canals in 3D printed tooth replicas (*n* = 18) was 10.02 ± 0.14 mm from the orifice to the apical foramen (AF) and 7.54 ± 0.17 mm from the furcation level to the AF, and the mean degrees of the canal curvature was 15.3 ± 1.0 degrees. The mean long and short diameters of the MM canals at the WL were 0.190 ± 0.019 mm and 0.128 ± 0.05 mm, respectively.

The high level of homogeneity of 3D printed tooth models among the 3 groups was confirmed by the measurements of root canal length, curvature, volume, surface area, and canal wall thickness (all *p* > 0.05) pre-instrumentation.

### Canal straightening

The measurement results of canal straightening of the MM canals after instrumentation with the 3 NiTi rotary systems are shown in Table 1. A statistically significant difference was detected among the 3 groups (*F* = 9.273, *p* = 0.002). The use of PTG resulted in significantly greater canal straightening than OS (*p* < 0.05) and TF files (*p* < 0.01), while no significant differences were obtained between OS and TF files (*p* > 0.05).

**Table 1:** Measurement results of canal straightening of the MM canals (*n* = 6 per group)

Instrument	Apical size	Straightening (°)
OS	#25	2.24 ± 0.31^b^
TF	#30	1.72 ± 0.93^b^
PTG	#30	3.25 ± 0.91^a^

Values with the different lowercase superscript letters along the same column are significantly different (*p* < 0.05). (OS—OneShape; TF—Twisted Files; PTG—ProTaper Gold)

### The root canal volume and surface area

The measurement results of the pre- and post-instrumentation volume and surface areas of the MM canals were summarized in Table 2. The PTG group obtained a significantly (*p* < 0.05) higher percentage increase in canal volume and surface area as compared with the OS and TF groups.

**Table 2:** Measurement results of the initial and post-instrumentation volume and surface areas of the MM canals (*n* = 6 per group)

NiTi System	Initial Volume (mm^3^)	Post-instrumentation Volume (mm^3^)	Volume increase (%)	Initial Surface (mm^2^)	Post-instrumentation Surface (mm^2^)	Surface increased (%)
OS	1.12 ± 0.10	2.32 ± 0.31^b^	107.9 ± 18.9^b^	11.51 ± 0.75	16.29 ± 0.79^b^	42.0 ± 10.1^ab^
TF	1.10 ± 0.14	2.07 ± 0.17^c^	89.1 ± 21.3^b^	11.89 ± 1.11	15.66 ± 0.87^b^	32.3 ± 10.9^b^
PTG	1.14 ± 0.08	2.71 ± 0.11^a^	138.5 ± 20.7^a^	12.03 ± 0.45	17.78 ± 0.42^a^	48.0 ± 6.3^a^
*F* value	0.180	23.090	9.050	0.658	13.72	4.342
*P* value	0.837	0.000	0.003	0.532	0.000	0.033

Values with the different lowercase superscript letters along the same column are significantly different (*p* < 0.05). (OS—OneShape; TF—Twisted Files; PTG—ProTaper Gold)

### The mesial and distal canal wall thickness at 1–3 mm below furcation

The measurement results of the minimum mesial and distal canal wall thickness pre- and post-instrumentation at the levels of 1, 2, and 3 mm below furcation are shown in Table 3. The remaining mesial and distal canal wall thickness post-instrumentation were significantly (all *p* < 0.05) lower in the PTG group as compared with the OS and/or TF groups for the MM canal.

**Table 3:** Measurement results of the mesial and distal minimum canal wall thickness pre- and post-instrumentation at the furcation levels (mm) (*n* = 6 per group)

Level	NiTi system	Pre-instrumentation minimum canal wall thickness			Post-instrumentation minimum canal wall thickness		
		MB	ML	MM	MB	ML	MM
1 mm below FL (Distal)	OS	1.25 ± 0.06	1.15 ± 0.09	1.22 ± 0.14	1.08 ± 0.08^b^	0.83 ± 0.06^b^	0.90 ± 0.11^b^
	TF	1.22 ± 0.05	1.12 ± 0.06	1.17 ± 0.08	1.04 ± 0.06^ab^	0.84 ± 0.05^b^	0.88 ± 0.07^b^
	PTG	1.25 ± 0.05	1.11 ± 0.05	1.20 ± 0.07	0.94 ± 0.10^a^	0.75 ± 0.05^a^	0.77 ± 0.05^a^
	*F* value	0.449	0.625	0.454	4.324	6.337	4.394
	*p* value	0.647	0.549	0.644	0.033	0.010	0.032
1 mm below FL (Mesial)	OS	1.22 ± 0.11	1.39 ± 0.06^*^	1.22 ± 0.05	1.17 ± 0.12^b^	1.26 ± 0.02^b*^	1.13 ± 0.07^b*^
	TF	1.24 ± 0.06	1.34 ± 0.05^*^	1.22 ± 0.07	1.13 ± 0.07^b^	1.23 ± 0.07^b*^	1.08 ± 0.08^ab*^
	PTG	1.21 ± 0.04	1.30 ± 0.09^*^	1.22 ± 0.05	0.96 ± 0.04^a^	1.14 ± 0.05^a*^	1.02 ± 0.04^a*^
	*F* value	0.203	2.690	0.025	11.52	10.95	4.110
	*p* value	0.819	0.100	0.975	0.000	0.001	0.038
2 mm below FL (Distal)	OS	1.15 ± 0.09	1.05 ± 0.10	1.01 ± 0.09	1.01 ± 0.09^b^	0.83 ± 0.09	0.76 ± 0.08^b^
	TF	1.11 ± 0.09	1.04 ± 0.08	0.99 ± 0.08	0.96 ± 0.10^b^	0.83 ± 0.07	0.82 ± 0.06^b^
	PTG	1.08 ± 0.07	1.06 ± 0.03	0.91 ± 0.07	0.81 ± 0.03^a^	0.74 ± 0.04	0.59 ± 0.06^a^
	*F* value	1.019	0.105	2.894	18.04	3.505	19.29
	*p* value	0.385	0.901	0.087	0.000	0.056	0.000
2 mm below FL (Mesial)	OS	1.09 ± 0.08	1.21 ± 0.04^*^	1.03 ± 0.05	0.99 ± 0.08^a^	1.06 ± 0.07^b*^	0.85 ± 0.03^b^
	TF	1.08 ± 0.11	1.19 ± 0.05^*^	1.01 ± 0.04	0.92 ± 0.08^ab^	1.03 ± 0.05^b*^	0.83 ± 0.04^ab^
	PTG	1.08 ± 0.04	1.18 ± 0.03^*^	0.99 ± 0.05	0.83 ± 0.04^a^	0.93 ± 0.03^a*^	0.77 ± 0.06 ^a*^
	*F* value	0.028	0.854	1.917	8.458	10.19	5.737
	*p* value	0.972	0.447	0.181	0.004	0.002	0.014
3 mm below FL (Distal)	OS	1.11 ± 0.08	0.94 ± 0.07	0.89 ± 0.07	0.97 ± 0.10^b^	0.80 ± 0.05^b^	0.75 ± 0.09^ab^
	TF	1.10 ± 0.04	0.92 ± 0.07	0.85 ± 0.09	0.96 ± 0.05^b^	0.79 ± 0.07^b^	0.79 ± 0.08^b^
	PTG	1.12 ± 0.07	0.90 ± 0.04	0.86 ± 0.05	0.84 ± 0.08^a^	0.69 ± 0.05^a^	0.65 ± 0.05^a^
	*F* value	0.115	1.645	0.876	5.042	10.33	5.626
	*p* value	0.893	0.226	0.437	0.021	0.002	0.015
3 mm below FL (Mesial)	OS	0.88 ± 0.09^*^	1.02 ± 0.04	0.84 ± 0.09	0.79 ± 0.10^b*^	0.82 ± 0.05^b^	0.67 ± 0.07^b*^
	TF	0.89 ± 0.06^*^	1.02 ± 0.04	0.84 ± 0.06	0.74 ± 0.03^ab*^	0.83 ± 0.04^b^	0.67 ± 0.03^b*^
	PTG	0.93 ± 0.07^*^	1.02 ± 0.07	0.83 ± 0.07	0.66 ± 0.05^a*^	0.72 ± 0.07^a^	0.57 ± 0.07^a^
	*F* value	0.861	0.012	0.145	5.699	7.654	5.275
	*p* value	0.443	0.988	0.486	0.014	0.005	0.018

Values with the different lowercase superscript letters along the same column are significantly different (*p* < 0.05); *Means statistically significant difference between mesial and distal sides for each instrument group (paired *t*-test, *p* < 0.05). (OS—OneShape; TF—Twisted Files; PTG—ProTaper Gold; FL—furcation level)

### Root canal transportation in the mesiodistal direction

Canal transportation in the mesiodistal direction at 1, 2, and 3 mm below furcation was summarized in Table 4. For the MM canal, significantly (*p* < 0.05) more pronounced distal transportation was detected in the PGT group than in the TF group at 2 mm below furcation, and significantly (*p* > 0.05) more pronounced mesial transportation was found in the TF group than in the OS group at 3 mm below furcation. For the MB and ML canals, however, there was no significant difference in canal transportation among the 3 instrument groups (all *p* > 0.05).

**Table 4:** Canal transportation at the levels of 1, 2, and 3 mm below furcation after instrumentation with 3 different NiTi rotary systems (mm) (*n* = 6 per group)

Level	Instrument system	Root canal		
		MB	ML	MM
1 mm below FL	OS	0.123 ± 0.067	0.191 ± 0.057	0.226 ± 0.079
	TF	0.077 ± 0.044	0.148 ± 0.095	0.155 ± 0.066
	PTG	0.056 ± 0.099	0.201 ± 0.152	0.223 ± 0.068
	*F* value	1.319	0.403	1.794
	*p* value	0.297	0.675	0.200
2 mm below FL	OS	0.042 ± 0.096	0.085 ± 0.065	0.065 ± 0.041^ab^
	TF	-0.009 ± 0.109	0.056 ± 0.056	-0.016 ± 0.027^b^
	PTG	0.013 ± 0.059	0.085 ± 0.052	0.109 ± 0.110^a^
	*F* value	0.464	0.42	5.369
	*p* value	0.638	0.663	0.017
3 mm below FL	OS	0.042 ± 0.077	-0.065 ± 0.079	-0.010 ± 0.027^a^
	TF	-0.013 ± 0.075	-0.053 ± 0.090	-0.111 ± 0.091^b^
	PTG	0.000 ± 0.078	-0.082 ± 0.036	-0.043 ± 0.033^ab^
	*F* value	0.848	0.235	4.732
	*p* value	0.102	0.793	0.026

Negative numbers represented deviation in the mesial direction, and positive numbers, in the distal direction. Canal transportation equal to 0 means that no transportation occurred. Values with the different lowercase superscript letters along the same column are significantly different (*p* < 0.05). (OS is OneShape; TF is Twisted Files; PTG is ProTaper Gold; FL is furcation level)

## Discussion

In this study, a permanent mandibular first molar with a typical MM canal (confluent category according to Pomeranz et al. classification [7]) was selected from a pool of extracted teeth, and the corresponding resin tooth replicas were fabricated via 3D printing technique. Regarding the pre-operative geometric measurement results, there was no significant (all *p* > 0.05) difference among the 3 groups (Tables 2 and 3), suggesting that the tooth replicas contain a unified canal shape. While previous ex vivo studies on root canal preparation often used extracted teeth, which have various root canal configurations, and as a result, standardization of teeth and anatomically balanced experimental groups could be hard to achieve. Simulated canals in resin blocks are used as a substitute for extracted teeth by many scholars because they have a standard canal length, curvature, and canal form, but they can’t fully reflect the anatomy of real human teeth [17, 26]. Our findings confirmed the previous reports [22, 24] that 3D printed tooth replicas have the advantages of consistency, reproducibility, and repeatability, and can be an ideal model to evaluate the performance of different instruments. They can simulate the clinical conditions and challenges of preparing an MM canal.

The root concaves of mandibular molars have attracted many scholars’ attention and interest as they are associated with the location of the danger zone and strip perforation during instrumentation [10, 27, 28]. As shown in Fig. 2, concaves were not only located at the distal side of the mesial root (furcation area), but also at the mesial side, forming a dumbbell-shaped root contour at the axial sections. As shown in Table 3, before instrumentation, only the ML canal was closer to the distal root surface at 1 and 2 mm below furcation (all *p* < 0.05), and the MM canal was at an equal distance (*p* > 0.05 for each level) from the mesial and distal root concaves. Instrumentation of the MM canal represents a challenge because it is often narrow, curved, and close to the root concave.

In recent decades, NiTi instruments have gained extensive acceptance among clinicians due to their improved flexibility and strength. The most concerned mechanical properties of NiTi files, which determine their shaping ability, include flexibility, torsional resistance, and flexural fatigue [29]. Flexibility is critical for maintaining the original canal shape and curvatures, while torsional resistance and flexural fatigue are associated with the occurrence of instrument separation, and cyclic fatigue is extremely important for NiTi rotary systems [29]. Meanwhile, enhanced flexibility can also decrease the cyclic stress of the file in the canal curvature, thereby reducing metal fatigue. The 3 NiTi instruments (OS, TF, and PTG) currently used are diverse in geometric design and metallurgy, and each of them has been reported to show good performance in curved canals owing to certain degrees of innovation in manufacturing [14, 18, 19]. However, it remains unclear whether they are suitable for shaping the MM canals, which are assumed to be more challenging than the MB and ML canals. In this study, instrument breakage (due to torsional overload or mental cyclic fatigue) was not detected in any groups, indicating each system has excellent fracture resistance and is safe in the MM canal.

Previous studies demonstrated that the geometric design, composition and thermomechanical treatment of the metallic alloy, and metal surface treatment may influence the file flexibility [29], while instrumenting a curved canal for a prolonged time with stiff files may cause canal straightening. In this study, we found in comparing both TF and OS systems, the use of PTG resulted in greater canal straightening (Table 1). PTG is manufactured with innovative gold heat treatment, which reportedly can increase the flexibility and resistance to cyclic fatigue [19], however, the current data indicates that the gold heat treatment could not offset the adverse effects caused by its geometric design and preparation fashion. The intrinsic curvature of the mesial root was mainly located at the coronal and middle root thirds (Fig. 3A), while instruments with a larger taper are associated with a larger diameter and stiffness of the instrument, especially at the coronal portion. Moreover, the crown-down technique adopted by PTG gravitated cervical “dentin” cutting, and the prolonged preparation time with multiple files (6 files were used) promoted canal straitening; while regarding TF, it produced the least canal straightening and more centered canal preparation, though the difference was not statistically significant as compared with OS. The excellent performance of TF can be attributed to the fact that TF files are manufactured by twisting rather than grinding, as twisting helps preserve the grain structure of metal and reduces the formation of microfractures [14, 29–31]. Moreover, R-phase heat treatment may optimize the mechanical properties of NiTi alloy, and special surface conditioning (surface deoxidation treatment) can further increase the flexibility and resistance to cyclic fatigue [14]. Regarding the geometric design, unlike PTG with a convex triangular cross-section, TF files have a triangular cross-section with constant tapers [14], and the reduced cross-sectional area and core area lead to the increased flexibility of TF. OS belongs to a single-file shaping system and adopts a constant taper of 6%. Although OS is made of a conventional austenite alloy, it possesses an innovative cross-section design (a triangle-shaped symmetrical three-cutting-edge in the tip area, a S-shaped symmetrical two-cutting-edge at the coronal area, and an asymmetrical progressively changing in the middle [18]), and the cross-sectional area or the core diameter is also less than that of a round triangle (PTG), which is favorable to enhance the file flexibility and decrease the canal straightening. Because OS only used 1 file with a tip size of 25 (which is one size smaller than the final file of TF and PTG), the cutting time was remarked reduced, and hence, OS was also excellent at preserving the original canal curvature as TF could do (the tip size is more important than the taper in affecting the apical transportation and canal straightening at the apical portion, which is not the focus of the current study).

Table 2 shows that the use of PTG resulted in significantly more resin removal, and yielded the largest post-instrumentation canal volume and surface area as compared with TF and OS,. This probably ascribes to the relatively larger taper and crown-down technique adopted by the PTG system. Coronal flare can remove cervical interferences around the orifice and encourage the straight-line access of the instrument to the apical portion of the root canal, which allows subsequent shaping files to prepare the apical portion with reduced taper lock effect and friction, thereby reducing the risk of instrument separation [32]. However, coronal flare is likely to modify the original canal shape, especially at the cervical portion [33]. The step of coronal flaring is emphasized by PTG system with SX file, is optional step for TF system and in the current study a TF 25/8% was used as the orifice opener, but such step not required by OS. Moreover, in this study, PTG system was used with 6 files and the final file had the largest tip size and taper (30/9%), while OS only had a single file with a smaller tip size and moderate taper (25/6%), and using only one file can remarkably reduce the preparation time and the amount of resin removal. TF was used up to a final file of 30/4%, and as shown in Fig. 1, its longitudinal section area is the smallest as compared with OS and PTG 30/9%, while PTG 30/9% as the final file has the largest longitudinal section area. This parameter is directly related to the post-instrumentation canal volume/surface area, which shares the same sequence of “PTG 30/9% > OS 25/6% > TF 30/4%”. As non-adaptive files, these instruments are prone to create a space representing their own shape in a straight canal, though in a curved canal, additional space may be created due to canal straightening. Collectively, our findings confirmed that the file geometry and preparation technique determined the amount and site of dentin/resin removal, and care should be taken to avoid excessive dentin sacrifice during instrumentation of the MM canal.

Measurement of pre-operative canal wall thickness at 1–3 mm below furcation indicates that the mean value decreased apically, and for the MM canal, it decreased from 1.22 mm to 0.85 mm at the distal side and from 1.22 to 0.83 mm at the mesial side. The use of PTG was associated with the thinnest remaining wall thickness as compared with the other 2 instruments, and for the MM canal, the mean distal wall thickness post-instrumentation was 0.77, 0.59 and 0.65 mm at 1, 2, 3 mm below furcation, respectively; while at the mesial side, the mean value was 1.02, 0.77, and 0.57 mm, respectively. This finding indicated that the danger zone could not only be positioned towards the distal side (furcation area), but also towards the mesial side (especially at 3 mm below furcation), which is in accordance with the results of 2 other micro-CT studies obtained by De-Deus et al. [27, 28] A recent study [34] using extracted mandibular molars showed that the fracture resistance of the mesial roots significantly decreased after the preparation of mesial canals with large-tapered instruments. Preparation of the MM canal further diminished the fracture resistance of the mesial roots, and the resultant fracture displayed a distinct pattern in the buccolingual plane [34]. This finding is in agreement with our result that instrumentation of the MM canal with large diameter/taper files (PTG) may cause excessive resin removal and thinner remaining canal wall thickness at the cervical aspect of the root, which may lead to root fragility. Dwivedi et al. [35] reported that the danger zone in longer mandibular molars was thinner than in shorter teeth, and the distal root concave was deeper in the longer teeth. The current study found the mean length of the MM canals was 10.02 mm from the orifice to the AF and 7.54 mm from the furcation level to the AF, which belongs to a short tooth. According to Fig. 1, we can obtain the corresponding file diameter and taper at each root level, to achieve a rough estimate of the file flexibility and cutting space. Theoretically, large-tapered instruments should be avoided or used with caution in preparing a longer MM canal to protect the danger zone.

The purpose of root canal shaping is to achieve a continuously tapered, funnel-shaped canal from orifice to apex and maintain the original canal curvature, thereby facilitating effective irrigation and 3D obturation [36]. Table 4 shows, despite the flexibility of the 3 NiTi instruments, in general, there was a tendency for the MM and ML canals to transport toward the distal aspect of the root at 1 and 2 mm below furcation (the transportation values are positive). Previous studies demonstrated that many factors may impact canal transportation, including the size and taper of the instrument, the metallurgical properties of the NiTi alloy, and the root canal anatomy [31]. We observed obvious distal canal transportation at the levels of 1 mm below furcation. While at the 2 mm below furcation, distal transportation was significantly reduced for each instrument group, and at the 3 mm below furcation, the transportation direction changed to the mesial side for the MM and ML canals (the transportation values are negative). These findings indicate that the canals were enlarged unevenly not only at the mesiodistal dimension but also at the coronal-apical dimension. In the canal curvature near the furcation levels, the instrument tends to recover its original linear shape and the point of inflection (the intersection point between the straight line and the central axis of the canal curvature) is located between 2 and 3 mm below furcation. Understanding this information may be useful for clinicians to reduce iatrogenic mishaps during the preparation of the MM canal. Generally speaking, the discrepancy in the amount of canal transportation among different instrument groups was not apparent. No significant difference was noted among the 3 instrument groups (all *p* > 0.05) in preparation of the MB and ML canals. But in the MM canal, significantly more transportation was detected only in the PTG group at 2 mm below furcation, and in the TF group at 3 mm below furcation (both *p* < 0.05). Collectively, the direction and amount of canal transportation is associated with canal straightening. Increasing the file flexibility and decreasing cervical instrumentation are strategies to reduce canal transportation and other iatrogenic errors near the danger zone.

This study has several limitations. First, all the 3D printed teeth are replicas of 1 human tooth, which may lead to selection bias. For example, in this study, the mean canal curvature of the MM canals was 15.3 degrees, which belongs to a moderate curvature, while other scholars frequently used severely curved canals (> 20 degrees) for preparation studies. Second, the operator had been familiar with the root canal configuration through high-resolution micro-CT images before instrumentation, which may decrease the operative difficulty as compared with the real clinical scenario. Finally, the hardness of the resin tooth models may differ from that of human teeth. Therefore, our conclusions derived from 3D printed tooth replicas required further verification by clinical practice.

## Conclusions

3D printed teeth are ideal models for evaluating the NiTi instruments’ shaping ability in the MM canal. Generally speaking, OS and TF systems performed similarly and both were appropriate for shaping the MM canal, while PTG may cause excessive and uneven “dentin”/resin removal and may lead to root fragility and procedural errors.

## Data Availability

All the datasets used and analyzed during the current study are available from the corresponding author on reasonable request.

## Abbreviations

3D: three dimensional
ANOVA: one-way analysis of variance
CEJ: cemento-enamel junction
CBCT: cone-beam computed tomography
micro-CT: micro-computed tomography
MB: mesiobuccal
ML: mesiolingual
MM: middle mesial
NiTi: nickel-titanium
WL: working length
